# A Molecular Dynamics Investigation of the Temperature Effect on the Mechanical Properties of Selected Thin Films for Hydrogen Separation

**DOI:** 10.3390/membranes10090241

**Published:** 2020-09-18

**Authors:** Sunday Temitope Oyinbo, Tien-Chien Jen

**Affiliations:** Department of Mechanical Engineering Science, University of Johannesburg, Gauteng 2092, South Africa

**Keywords:** molecular dynamics, nanoindentation test, mechanical properties, plastic deformation

## Abstract

In this study, we performed nanoindentation test using the molecular dynamic (MD) approach on a selected thin film of palladium, vanadium, copper and niobium coated on the vanadium substrate at a loading rate of 0.5 Å/ps. The thermosetting control is applied with temperature variance from 300 to 700 K to study the mechanical characteristics of the selected thin films. The effects of temperature on the structure of the material, piling-up phenomena and sinking-in occurrence were considered. The simulation results of the analysis and the experimental results published in this literature were well correlated. The analysis of temperature demonstrated an understanding of the impact of the behaviour. As the temperature decreases, the indentation load increases for loading and unloading processes. Hence, this increases the strength of the material. In addition, the results demonstrate that the modulus of elasticity and thin-film hardness decreases in the order of niobium, vanadium, copper and palladium as the temperature increases.

## 1. Introduction

Over the last few years, large digital computers have been used to study different facets of molecular dynamics in liquids, gasses and solids [1]. In the determination of solid material’s behaviour, crystalline structure and atomic arrangement are important. A thin layer of the metal composite membrane such as palladium (Pd), vanadium (V), copper (Cu) and niobium (Nb), which are coated on vanadium substrate, is of great importance in fuel reforming (hydrogen separation) processes since the much lower cost of vanadium (V), copper (Cu) and niobium (Nb) materials than that of pure palladium or palladium alloy allows the membrane to have enough strength and thickness for the required operating pressure and temperature. The thin films mechanical properties, which are not the same with their mass samples, should be deduced precisely for the superior performance of the micro-equipment [2,3]. As the thickness of the thin films reduces in industrial operations, their mechanical characteristics are increasingly difficult to calculate. Therefore, the standard approaches used in mass samples are not ideal for thin films [4]. Environmental conditions of the films such as temperature and moisture can have a direct influence on their mechanical properties [4,5,6]. Few methods exist to measure the surface strength of thin films, which in recent years have gained greater attention from the nanoindentation experiment. The main objective of this method is to calculate the strength and elasticity modulus of several materials. Many investigators have used experimental and theoretical methods for extracting the mechanical properties of coatings in nanoindentation from the film–substrate systems [5,7,8,9]. As there are some limitations on the nanoindentation equipment, such as machine resolution, signal-to-noise ratio and tip rounding effects, it is fairly difficult to obtain practical experimental results at indentation depths under 10 nm [10,11]. In the conducted experiments, the indentation depth is usually limited to a small portion of the thin film thickness, typically 10%, to avoid the effect created by the substrate on the measured parameters. The thin film is so small in thickness that 10% of its thickness is in a range in which there may be problems with the effects of the indentation dimension and the roughness of the surface. It is thus critical that the mechanical characteristics of thin films are determined using new methods across several indentation depths, which are not sensitive to physical properties such as piling-up phenomena, sinking-in occurrence and roughness of the surface [12,13].

Atomistic simulations like molecular dynamics (MD), a methodology of detailed atomic material modelling, have provided useful information on the atomic structure, deformation of subsurface and material defect dynamics. The MD methodology measures the motion of individual atoms/molecules in material simulation and explains how their positions, speeds and orientations change over time. MD simulations were subsequently used extensively for the study of nanoindentation and the exploration of nanoscale and frictional mechanisms [4,14,15].

Several researchers have recently used MD models to test the mechanical characteristics of thin films with nanometric indentation. For example, Shi and Falk [16] used models of MD for the simulation of the crystalline and the thin metal film nanoindentation process as well as for the analysis of structural transformation and the position of atoms during indentation. Peng et al. [17] adopted molecular dynamics simulation for a three-dimensional nanoindentation test for the silicon substrate coated with aluminium particles. The copper cluster deposition on silicon substrates was simulated by Hwang et al. [18] using molecular dynamics modelling.

Therefore, this study aimed to use molecular dynamics simulation techniques to investigate the effect of temperature on the mechanical properties of the selected thin films of palladium, vanadium, copper and niobium deposited on the vanadium substrate at 0.5 Å/ps loading rate of nanoindentation process.

## 2. Material and MD Simulation Methods

**Figure 1:**
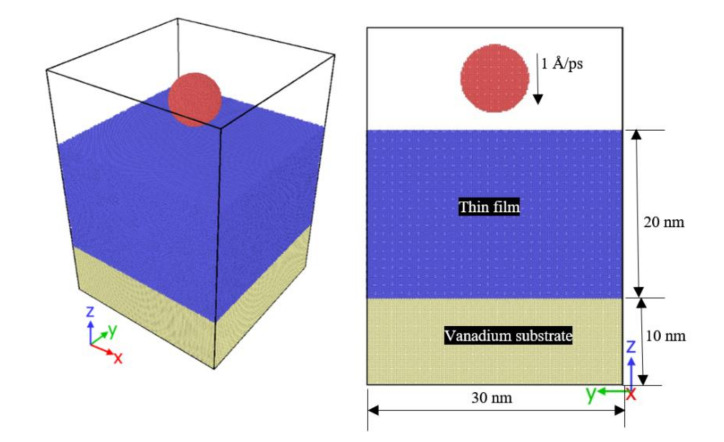
Simulation model snapshot.

For this method to be simulated, 3-dimensional sample models are built using the LAMMPS (Large Atomic/Molecular Parallel Simulator) [19] open-source program for simulating molecular dynamics. The hybrid interatomic potential of Lennard-Jones [20], Tersoff [21] and the embedded atom method (EAM) [22] has been used to describe atomic interactions. Although EAM potential is possible to determine the potential for interaction of the palladium, vanadium, copper and niobium, Lennard-Jones potential is used for interactions between different metals and Tersoff potentials are used to form the interaction between carbon atoms (diamond indenter). As the Figure 1 shows, MD models investigated in this study consisted of the spherical diamond indenter (10 nm diameter), the vanadium substrate (30 nm × 30 nm × 10 nm) and a CGDS thin film (30 nm × 30 nm × 20 nm) coated on the substrate. Thin-film coatings of palladium and copper have FCC lattice structures, and thin-film coatings of vanadium and niobium have a BCC lattice structure, which is oriented in the plane (1 0 0). These particles were coated on the surface of the substrate of vanadium oriented in the (1 0 0) direction. Periodic boundary conditions were used in the x and y directions and non-periodic boundary conditions in the z-direction. The integration time step of 0.5 femtoseconds (fs) was used. For energy minimization at zero temperatures, the systems were subject to a minimum 10 ps simulation time. The Nosé–Hoover thermostat [23] was adopted to maintain the temperature of the system at 300 K to both equilibrate the substrate and the thin films for 10 ps with LAMMPS NVE ensemble. The thermal bath was removed, and the whole system was supposed to be insulated thermally for 10 ps. To boost simulation measurement efficiency, the cutoff distance was adopted. To prevent contact between indenter and specimen, the indenter has been placed one interatomic cutoff radius above the substrate surface. For the displacement control technique, the indenter is inserted at a constant speed in the free surface of the coating. The loading rate for indentation is 0.5 Å/ps, and the calculation time is rational. The maximum depth of indentation is approximately 3 nm. The indenter is kept at maximum depth for about 10 ps to allow the model for re-equilibration. Table 1 explains the simulation conditions for the study.

## 3. Results and Discussion

### 3.1. Effect of Temperature Distribution during Nanoindentation Test

**Figure 2:**
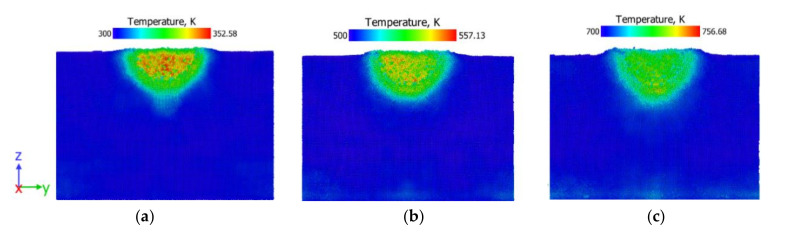
Cross-section configurations of vanadium thin film at (**a**) 300 K, (**b**) 500 K and (**c**) 700 K at 0.5 Å/ps loading rate.

**Figure 3:**
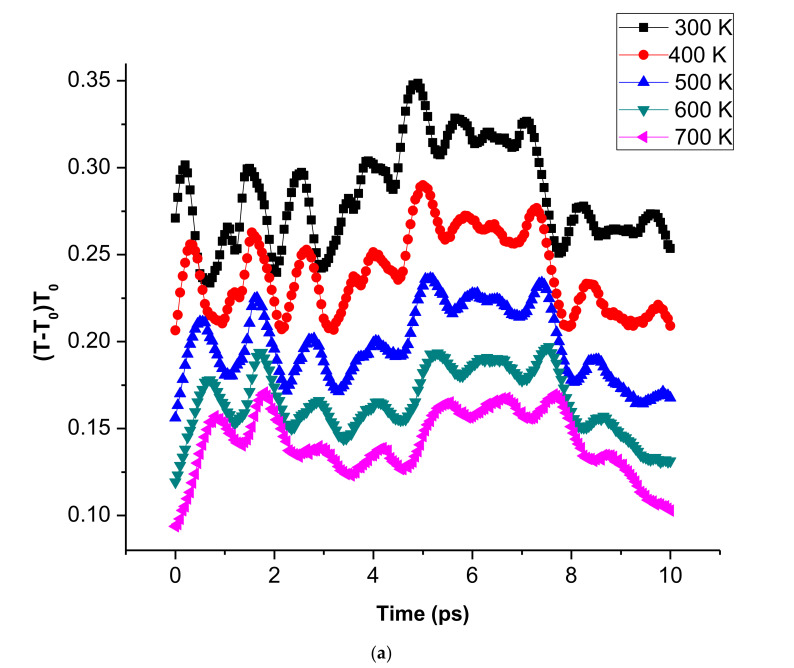

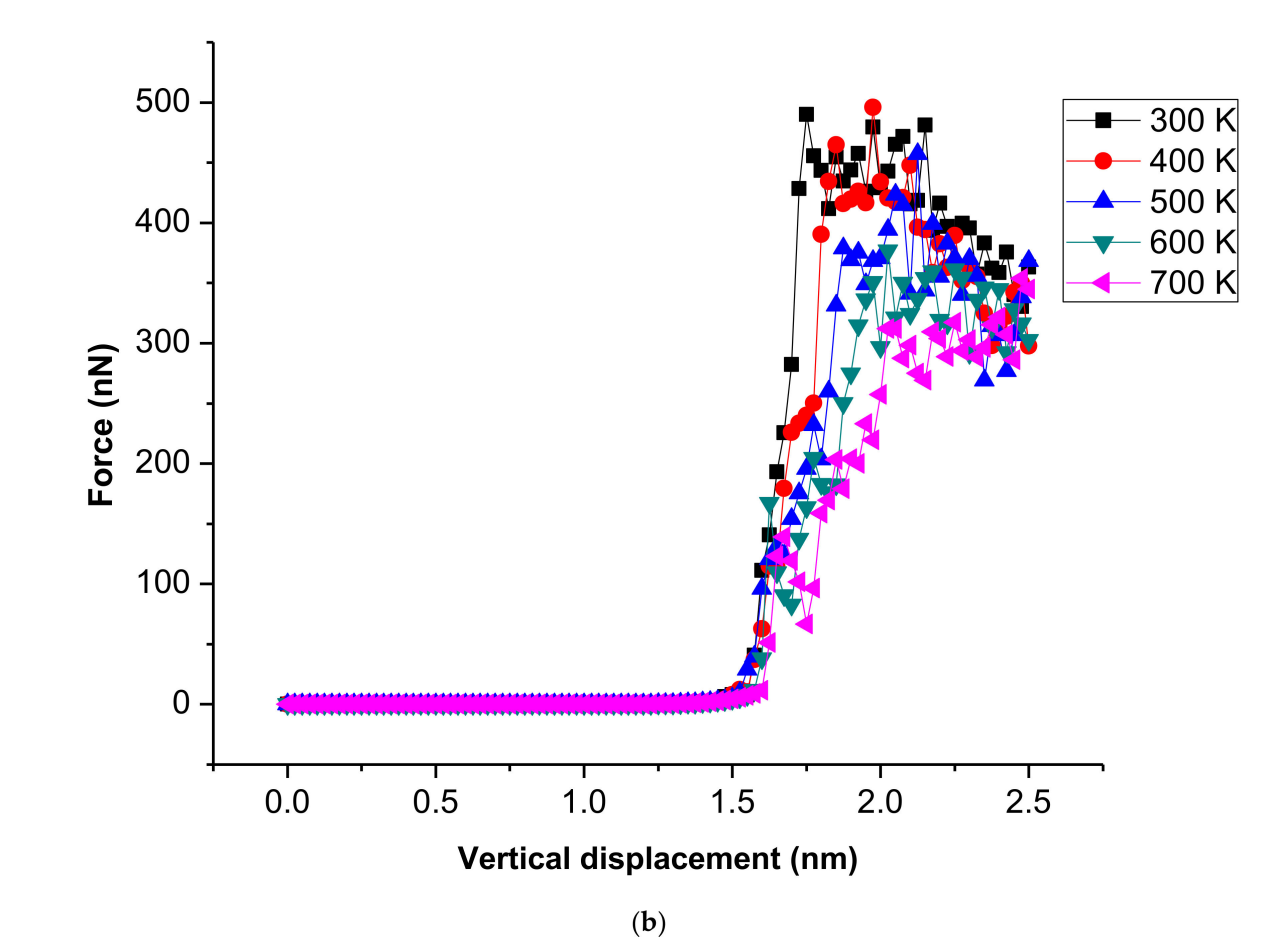
(**a**) The resultant computed temperature distributions on vanadium (V) thin film at 0.5 Å/ps loading rate for different temperatures during the loading process and (**b**) the Force-displacement curves during nanoindentation on niobium (Nb) thin-film during 0.5 Å/ps loading rate at different temperatures.

Figure 2 displays the thin-film model’s temperature evolution snapshots, indented for various temperatures at 0.5 Å/ps loading rates. The adiabatic temperature in this area increases with the dissipation of plastic work locally along the route of indentation. When the temperature increases, the deformation experienced by the thin film increases. As the temperature rises for loading and unloading processes (Figure 3a), the resultant temperature in the thin films decreases.

Figure 3b illustrates the load-displacement curve at load levels of 0.5 Å/ps at different temperatures. The load-displacement curves show the occurrence of a decreasing force with increasing thin-film temperature. The indenter load decreases at a constant depth as the temperature increases. This was because of thermal softening. The indentation thermal softening occurred as a result of the material dislocation propagation that easily slips at high temperature or high kinetic energy. In addition, when nanoindentation was carried out with high temperatures, the behaviour of thermal softening led to a Young’s modulus reduction in the thin film. Due to the plastic deformation in the depth of indentation, Young’s modulus reduction resulted from the temperature increase is slightly higher. The elastic regeneration at higher temperatures is also smaller. Due to the plastic deformation at the indentation depth, the reduction of Young’s modulus can be seen to increase slightly with rising temperature. The elastic recovery at higher temperatures is also smaller. There is a consistency of this behaviour to macrobehaviour [24]. The interaction binding energy of the workpiece decreases when the temperature increases, and thus resulting in the decrease of material hardness from the microscale level. The Lebedev et al. [25] microscale experimental investigation confirms this phenomenon where the elastic modulus decreased when the temperature rose in sub-microcrystalline copper. The increase in temperatures causes the force-displacement curves to fluctuate as a result of vibration of atoms and the nucleation of voids at the depth of indentation within the substrate.

### 3.2. Effect of Indentation Loading Rate on the Thin Film Mechanical Properties

During the MD simulation of nanoindentation test, the continuous force-displacement data obtained by the complete process of loading and retraction were used to extract the thin films mechanical properties. The Sneddon relationship (Equation (1)) can be used to measure the effective elasticity modulus ($\gamma_{e}$) [7].

(1) $$\frac{1}{\gamma_{e}}=\frac{\gamma_{i}\left(1-\gamma^{2}\right)+\gamma \left(1-v_{i}^{2}\right)}{\gamma \gamma_{i}}$$

where v and γ are the Poisson’s ratio and elasticity modulus, respectively, of the sample, $v_{i}$ and $\gamma_{i}$ are the Poisson’s ratio and elasticity module, respectively, of the indenter, and $\gamma_{e}$, the effective nanoindentation test module of elasticity, which can be obtained using Equation (2) [7]:

(2) $$\gamma_{e}=\frac{S_{t}\sqrt{\Pi }}{2\sqrt{A}}$$

where A and $S_{t}$ are the cross-sectional area of the indentation depth and film stiffness, respectively. $S_{t}$ is extracted from the force-displacement curve as the computed slope of the unloading curve and is given as:

(3) $$S_{t}={\left|\frac{dL}{dh}\right|}_{L=F_{max}}$$

The maximum force is denoted as by $F_{max}$ during the nanoindentation calculation. Two parameters should be taken into account when calculating the surface area of the indentation depth within the thin film: the indenter geometry and the depth of indentation after the unloading process.

**Figure 4:**
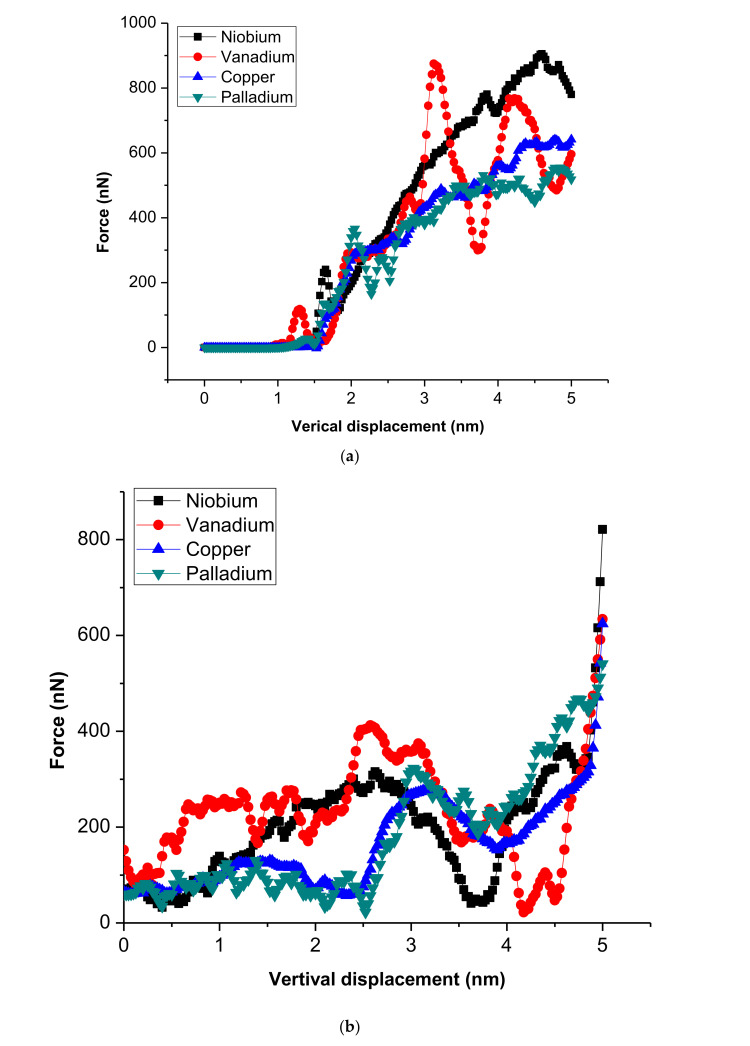
Force-displacement curves for (**a**) loading and (**b**) unloading process during the nanoindentation test of the thin films at 0.5 Å/ps loading rate.

**Figure 5:**
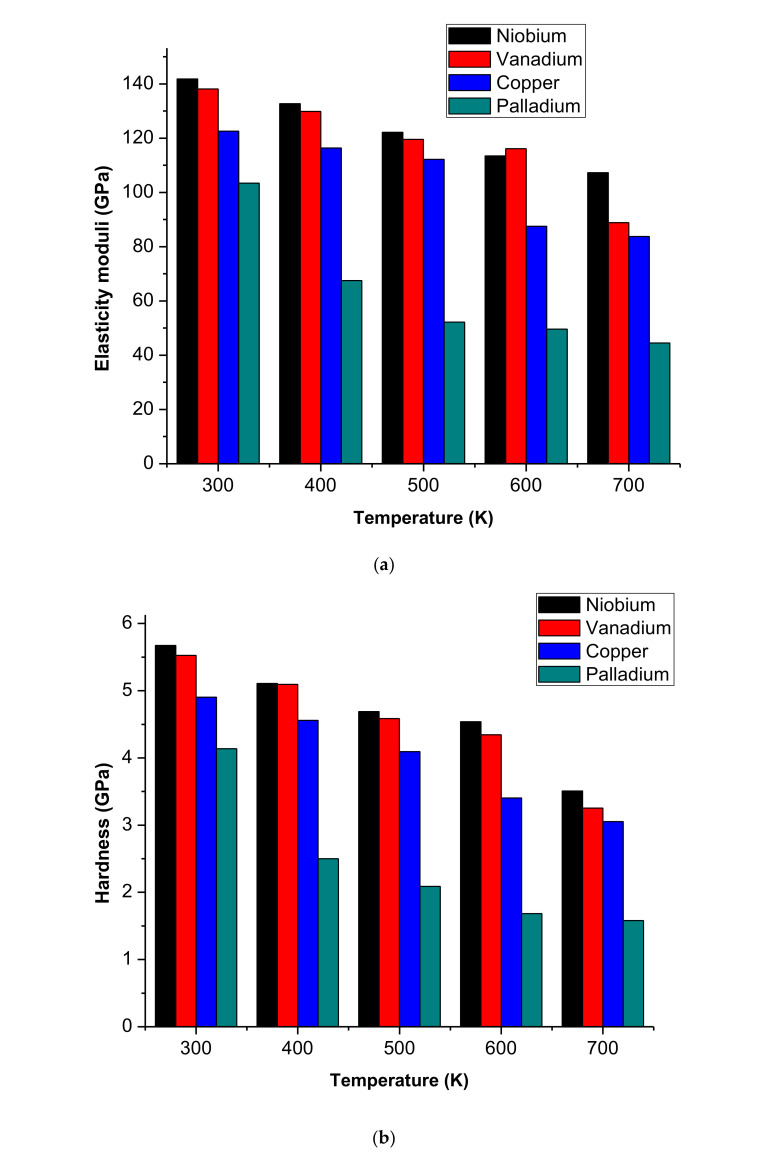
(**a**) The elasticity moduli versus temperature. (**b**) Hardness values versus temperature of the thin film coatings obtained from molecular dynamic (MD) simulation.

The force-displacement curves obtained from the nanoindentation test during molecular dynamics simulation at a temperature of 700 K for various thin films are shown in Figure 4. As the figure shows, the maximum force of indentation decreases in the order of niobium, vanadium, copper and palladium. The strength of the thin film decreases with decreasing maximum force. Therefore, the interface of the sample that surrounds the indenter is usually drawn inwards (sunk in)/outwards (piling-up). This indentation process involves considerable plastic deformation. The piling-up phenomena and sinking-in occurrence are regarded as errors in this operation and can influence the cogency of the results [12]. The primary reason behind the piling-up phenomena is that the indented region is subjected to plastic deformations. The plastic deformation increases as the temperature increases around the indenter because of the friction between the indenter and the film surface and the interactions among atoms. The nanoindentation test is a continuous operation, leading to uncontrollable temperature changes in the indenter, which exacerbate these errors. The elasticity modulus values from Equation (2) are measured, and the results are shown in Figure 5a. The results demonstrated the variation of the temperatures in the thin film (due to discrepancies between atomic layer interactions and atoms of the indenter) and the differences among atoms of each thin film cohesive energies.

Comparative values in Figure 5a indicate that the modulus of elasticity decreases as the temperature increases. This decrease is because of the increase in the ratio of plastic deformation against elastic deformation in the indented region. It must be noted that the temperature increases by increasing the number of voids and the defects in the structure of the film, leading to the expansion of the amorphous portion of the structure of the crystalline film.

**Figure 6:**
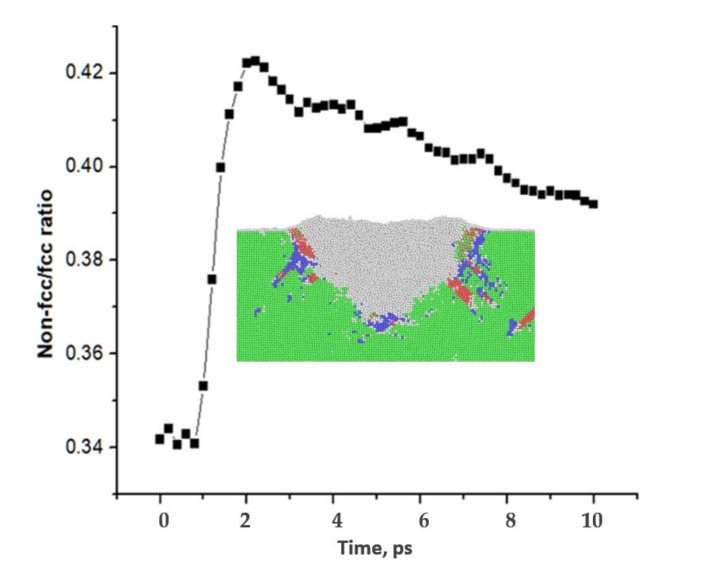
Structural transformation (defect nucleation) at the depth of indentation showing non-fcc/fcc structures ratio with time. Inset: cross-section of Pd thin film (green: fcc structure; grey, blue and red: non-fcc structure).

A sudden fall in the indenter force detects plastic deformation of material during displacement-controlled indentation simulations. The sudden fall in the indenter force is a result of the nucleation of the defects or phase transition. To define these defects or structural transformations, a quantitative ratio between non-fcc structures and fcc structures was used in the palladium thin film counterparts (see Figure 6); the sum of non-fcc structures increased with increasing impact time and latter decreased due to the gradual rearrangement (elastic recovery) of the fcc structure at the depth of indentation.

A simple equation (Equation (4)) [7] is used to measure material hardness *H*, which is deduced by the ratio of the maximum load ($L_{max}$) for the projected contact cross-section area.

(4) $$H=\frac{L_{max}}{A}$$

By the geometry of the indenter tip, which is to be expressed as the expected contact area, *A* can roughly be determined as [7]:

(5) $$A=\;\frac{3\sqrt{3}}{2}h^{2}$$

where *h* is the indentation depth.

The results of the simulation were substituted into Equation (4), and the hardness values of thin films were obtained. Figure 5b shows the determined hardness values for niobium, vanadium, copper and palladium at different operating temperatures. Temperature affected both parameters used to calculate the hardness of the material. The maximum load ($L_{max}$) decreased by increasing the temperature and the projected area of contact increased. The explanations are the same as for the modulus elasticity mentioned earlier.

### 3.3. Comparison with Previous Experimental and Numerical Results

Because of the limited experimental data with the same process parameters used in the present molecular dynamics simulation as compared with the one presented in the literature, current simulation findings for Cu are compared with the Young’s modulus of MD simulation and experimental study by Ayatollahi et al. [25] and Lebedev et al. [4] respectively. Lebedev et al. obtained approximately 116–126 GPa for Cu Young’s modulus (with temperature variance between 20 and 300 °C). Ayatollahi et al.’s MD simulation result predicted Cu Young’s modulus (with temperature variance between 193 and 793 K) as 54–153 GPa, while the current MD simulations were found to be 88–122 GPa (with temperature variance between 300 and 700 K). Hung et al. [26] reported material hardness of Cu to be around 0.9–4.4 GPa (while temperatures were varied from −190 to + 60 °C), and hardness of 3.0–4.9 GPa were recorded in the current simulations (with temperature ranging from 300 to 700 K). In our simulation, the elasticity modulus and hardness values are significantly similar to those stated in the literature. The inconsistency in the values can, however, be caused by the difference in scale between the simulation and the experiment, i.e., nanoscale and microscale. The defect effect on the deformation mechanisms of the material differs at various scales.

## 4. Conclusions

This research simulated nanoindentation processes using a molecular dynamics approach for determining the temperature effects on palladium, vanadium, copper and niobium CGDS thin film coatings on the mechanical properties. The effects of temperature on the structure of the material, piling-up phenomena and sinking-in occurrence were simulated. The results suggest that the analysis of resultant temperature showed an understanding of deformation behaviour during the nanoindentation test. As the temperature decreases, the indentation load increases for loading and unloading processes. Hence, this increases the strength of the material. In addition, the results demonstrate that the modulus of elasticity and thin-film hardness decreases in the order of niobium, vanadium, copper and palladium as the temperature increases. As the temperature increases, the elasticity modulus and hardness decreases. The decline in the mechanical properties is due to increase in voids nucleation and defects in the crystalline structure of the thin film as well as an increase of plastic deformation in relation to elastic deformation. The elastic recovery is reduced by increasing the temperature.

## Figures and Tables

**Table 1:** Schematic calculation plan used in the molecular dynamic (MD) simulation.

Materials	Thin Films	Rectangular Block—30 nm × 30 nm × 20 nm Palladium (1,237,288 Atoms), Vanadium (1,306,866 atoms), Copper (1,515,580 Atoms) and (Niobium (1,002,001 Atoms)
	Substrate	Rectangular block—30 nm × 30 nm × 10 nm Vanadium (663,433 atoms)
	Indenter	Spherical diamond indenter (diameter: 10 nm), 23,669 atoms
Operating conditions	Duration of simulation	10 ps (10,000 fs)
	Timestep	0.5 fs (0.0005 ps)
	Loading rate	0.5 Å/ps
	Potential used	Lennard-Jones, Tersoff and EAM
	Preheating temperature	300, 400, 500, 600 and 700 K
	Boundary condition	p p s
